# Nano Zero Valent Iron (nZVI) as an Amendment for Phytostabilization of Highly Multi-PTE Contaminated Soil

**DOI:** 10.3390/ma14102559

**Published:** 2021-05-14

**Authors:** Maja Radziemska, Zygmunt M. Gusiatin, Jiri Holatko, Tereza Hammerschmiedt, Andrzej Głuchowski, Andrzej Mizerski, Iwona Jaskulska, Tivadar Baltazar, Antonin Kintl, Dariusz Jaskulski, Martin Brtnicky

**Affiliations:** 1 Institute of Environmental Engineering, Warsaw University of Life Sciences, Nowoursynowska 159, 02 776 Warsaw, Poland; 2 Faculty of Geoengineering, University of Warmia and Mazury in Olsztyn, Słoneczna St. 45G, 10 719 Olsztyn, Poland; mariusz.gusiatin@uwm.edu.pl; 3 Department of Agrochemistry, Soil Science, Microbiology and Plant Nutrition, Faculty of AgriSciences, Mendel University in Brno, 61 300 Brno, Czech Republic; jiri.holatko@mendelu.cz (J.H.); tereza.hammerschmiedt@mendelu.cz (T.H.); tivadar.baltazar@mendelu.cz (T.B.); kintl@vupt.cz (A.K.); martin.brtnicky@seznam.cz (M.B.); 4 SGGW Water Centre, Warsaw University of Life Sciences—SGGW, 02 787 Warsaw, Poland; andrzej_gluchowski@sggw.edu.pl; 5 The Main School of Fire Service, Slowackiego 52/54, 01 629 Warsaw, Poland; amizerski@sgsp.edu.pl; 6 Department of Agronomy, Faculty of Agriculture and Biotechnology, University of Science and Technology, 7 Prof. S. Kaliskiego St., 85 796 Bydgoszcz, Poland; jaskulska@utp.edu.pl (I.J.); darekjas@utp.edu.pl (D.J.); 7 Agricultural Research, Ltd., Zahradní 1, 664 41 Troubsko, Czech Republic; 8 Institute of Chemistry and Technology of Environmental Protection, Faculty of Chemistry, Brno University of Technology, 61200 Brno, Czech Republic

**Keywords:** soil degradation, potentially toxic elements, phytoremediation, nanoremediation

## Abstract

In recent years, a lot of attention has been given to searching for new additives which will effectively facilitate the process of immobilizing contaminants in the soil. This work considers the role of the enhanced nano zero valent iron (nZVI) strategy in the phytostabilization of soil contaminated with potentially toxic elements (PTEs). The experiment was carried out on soil that was highly contaminated with PTEs derived from areas in which metal waste had been stored for many years. The plants used comprised a mixture of grasses—*Lolium perenne* L. and *Festuca rubra* L. To determine the effect of the nZVI on the content of PTEs in soil and plants, the samples were analyzed using flame atomic absorption spectrometry (FAAS). The addition of nZVI significantly increased average plant biomass (38%), the contents of Cu (above 2-fold), Ni (44%), Cd (29%), Pb (68%), Zn (44%), and Cr (above 2-fold) in the roots as well as the soil pH. The addition of nZVI, on the other hand, was most effective in reducing the Zn content of soil when compared to the control series. Based on the investigations conducted, the application of nZVI to soil highly contaminated with PTEs is potentially beneficial for the restoration of polluted lands.

## 1. Introduction,

Industrial and civilizational progress, in addition to the advantages that it goes hand in hand with, contributes to the degradation of the natural environment, which largely pertains to soil [1,2]. Soil is one of the main elements of the natural environment determining, among others, the chemical composition of plants. Contamination with potentially toxic elements (PTEs), which may be derived from various anthropogenic activities, warrants particular attention [3,4]. The pressures induced on the soil environment that are connected with such activities strongly impact the quality of life, especially that observed in urban, industrial as well as agricultural areas [5,6,7].

The cultivation of degraded areas is a very significant issue due to the constantly increasing shortage of space. The newest findings point to the fact that there are over 10 million highly contaminated areas, with PTEs comprising over 50% of these contaminants [8]. Because these contaminants and their negative effects are increasingly global in their nature, it is thus necessary to develop effective methods for the protection and reclamation of contaminated areas.

Among the biological methods drawing much attention is phytoremediation [9]. Due to the complexity of processes accompanying phytoremediation, especially the means by which toxic compounds are taken up and eliminated by plants (accumulation, sequestration, degradation, transpiration), we can identify a few types of these methods. These include phytoextraction, phytostabilization, phytodegradation, phytovolatilization, and rhizodegredation [10]. Phytostabilization relies on contaminated areas being colonized by plants which tolerate high concentrations of PTEs; it also prevents the spread of contaminants throughout the environment [11]. Aided phytostabilization is a relatively newly applied technique for the bioremediation of the environment. This method relies on immobilizing, among others, PTEs found in the soil, with adequately selected phytostabilizing plants, and on soil’s supplementation with various kinds of soil additives [12]. The immobilization of PTEs in this case relies on a few processes, which can include adsorption as well as accumulation in the roots, adsorption on the surface of the roots, or transformation within the rhizosphere into poorly soluble substances [13]. Aided phytostabilization focuses, above all, on the below-ground zone of plants [14]; the chemical and biological processes taking place in this area contribute to the contaminants being retained in their tissues. The idea behind the process involves using plants to accumulate PTEs and to block them from penetrating the deeper layers of the soil profile [15].

Seeking new, safe, and more effective materials which can be used as soil additives which aid the processes of immobilizing contaminants in the soil is of key importance when it comes to intensifying the processes of immobilizing PTEs in the soil and improving its quality. Upon analyzing the findings from recent years, the use of nanoparticles of zero valent iron (nZVI) in aiding the remediation and phytoremediation processes of soils contaminated with PTEs may offer a new promising alternative in comparison to commonly applied soil additives [16,17,18,19,20]. Above all, it is thanks to this result the reduction and catalytic properties of nZVI, that it is possible to apply them practically in technologies for the reclamation of soils and groundwater [21]. The nZVI particles are active, above all, in oxidation–reduction reactions and can react with dioxygen dissolved in water, which can be used in the reduction of various contaminants occurring in the water–soil environment [22]. A significant drawback of nZVI particles—their tendency to aggregate resulting in the limitation of the distance of migration and thus decreased effectiveness—should, however, be mentioned [23,24]. The novelty of this work is connected with focusing on the possibility of using the properties of nZVI to improve the effectiveness of PTE immobilization in the aided phytostabilization technique in soils which were derived directly from real contaminated areas and which were a hotspot. The objective of this study was to evaluate the effect of an nZVI amendment on the enhanced phytostabilization of soils highly contaminated with PTEs by determining the plant yield, the chemical composition of the plant biomass accounting for the aboveground parts and roots, and the selected physical and chemical properties of the soil.

## 2. Materials and Methods

### 2.1. Study Site and Soil Sampling Procedure

**Figure 1:**
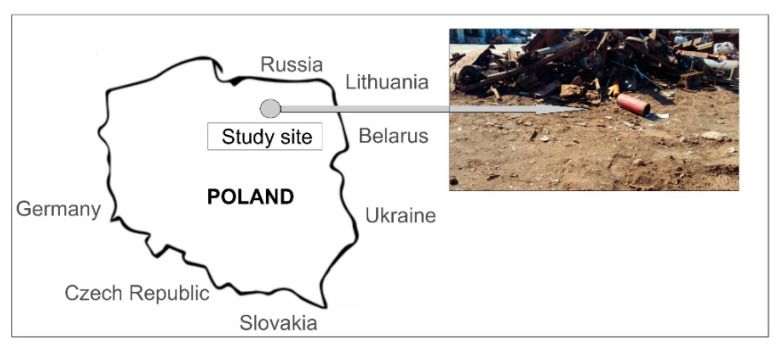
Localization of the study site in Northeastern Poland.

The study was carried out on soil from an area of steel disposal dumps in Northeastern Poland (Figure 1). The research area covered soils on which ferrous and non-ferrous (brass, aluminum, copper, zinc, and lead) waste of used batteries and accumulators had been stored for over 70 years. Due to the fact that the waste had been stored directly on the soil, high concentrations of PTEs were noted. Their values in soil subjected to analyses diverge greatly from the standards currently in force in Poland [25] when it comes to PTEs’ contents in the near-surface layer of the land. This is particularly observable for Pb and Zn where the permissible limit was exceeded multifold. Selected physical and chemical properties of the soil are presented in Table 1.

In the oldest part of the studied area, with a surface of 300 m^2^, the places where soil samples were taken for the studies were selected based on a point-based identification of the state of the soil environment. Surface soils (0–25 cm) were sampled with a stainless-steel shovel. At each point, 20 individual samples were collected, which were then mixed and treated as an average sample. All of the collected soil samples were carefully transferred to clean polyethylene bags before being transported to the laboratory. They were then air-dried at room temperature, sifted through a 2 mm sieve, and stored in a 4 °C refrigerator.

### 2.2. Experiment Description

A greenhouse experiment was conducted as described by Radziemska et al. [26]. In total, 5.0 kg of polyethylene pots was used in the experiment. Each pot was filled with a mixture of the soil and nZVI at a dose of 2.0% (w/w) and mixed thoroughly. The mass of nZVI used in the experiment was chosen as cited in Baragaño et al. [27,28]. Non-amended pots containing only the contaminated soil were used as controls. nZVI (iPutec GmbH & Co. KG, Rheinfelden, Germany) was characterized by a strongly alkaline pH of 11.45, average size of 50 nm, and it contained 92% Fe mixed with: C 2.8–3.2%, Si 1.8–2.1%, P 0.04–0.4%, Cr 0.05–0.4%, Ni 0.05–0.3% and Al 0.01–0.1% [29]. The soils with nZVI in individual pots were then kept in a dark room for 2 weeks to ensure stabilization under natural conditions prior to planting. Following this, plant seed mixtures (*Lolium perenne* L. cv. Nur, *Festuca rubra* L. cv. Aido) were sown into pots for 5 g seeds per pot. Experiments were carried out in triplicate. During the experiment, deionized water was supplied to bring the soils to approximate 60% of the maximum water holding capacity of the soil. The greenhouse was maintained at an average temperature of 26 ± 3 °C during the day (14 h) and 16 ± 2 °C at night (10 h). The plants were harvested for 67 days after planting, then weighed and separated into aboveground parts and roots, which were carefully washed with deionized water.

### 2.3. Soil Analysis

Following the pot experiment, soil samples were air-dried and sieved through a 2 mm mesh and homogenized in order to carry out the analytical procedures. Soil pH was determined using a pH electrode in a 1:2.5 water to soil extract using a pH-meter HI 221 (Hanna Instruments, Washington Hwy Smithfield, RI, USA). To determine the total contents of PTEs (Cu, Ni, Cd, Pb, Zn, Cr) in the soil, the samples were mineralized in a mixture of 36% HCl (9 mL), 65% HNO_3_ (3 mL), and 30% H_2_O_2_ (1 mL) in a microwave oven MARSXpress (CEM Corporation, Matthews, NC, USA). The PTEs’ contents were measured via flame atomic absorption spectrometry (Varian, Mulgrave, Australia, AA28OFS spectrometer) using a sample introduction pump system to enable automatic sample dilution. The quality of the analyses was assessed using reference material (CRM 142 R), and the obtained recoveries ranged from 95% to 101%. For the chemical analyses, ultra-pure water of 18 MΩ cm resistivity was taken from a Millipore System, (MilliQ Integral; Merck MilliporeCorp., Kenilworth, NJ, USA).

### 2.4. Analysis of Plant Biomass

At the end of the experiment, aboveground plant biomass was measured, and biomass was carefully washed with deionized water. Before the chemical analysis, the plants were powdered using an analytical mill (Retsch, ZM300, Hann, Germany). Aboveground parts and roots samples were mineralized in concentrated HNO_3_ and 30% H_2_O_2_ using a microwave oven MARSXpress (CEM Corporation, Matthews, NC, USA). The digested samples were then cooled down, filtered through Whatman 42 filters into a 100 mL volumetric flask, and filled up to volume with ultra-pure water. PTE concentrations in the filtrates were determined by flame atomic absorption spectrometry (Varian, AA28OFS, Mulgrave, Australia).

### 2.5. Statistical Analysis

All analyses were performed using freely available statistical software R, version 3.6.3. [30]. To model the relationship between the total contents of PTEs (Cu, Ni, Cd, Pb, Zn, Cr) in the soil and in the plant biomass dependening on the selected treatments (control, nZVI), a one-way analysis of variance (ANOVA) type I (sequential) sum of squares at a significance level of 0.05 was used. To calculate the factor level means, “treatment contrasts” were used, and to determine the difference among factor level means, Tukey’s Honestly Significant Difference (HSD) Test was used. After all statistical analyses were performed, the assumptions of selected models were checked with the help of appropriate statistical tests and different diagnostic plots.

## 3. Results and Discussion

### 3.1. Initial Soil Characterization

Soil used in the experiment was characterized by an alkaline pH of 7.45 ± 0.4, low organic matter content (1.3 ± 3.2%), and a relatively high cation-exchange capacity (50.7 ± 3.4 cmol/kg) and was like loamy sand in texture (71.1% sand, 27.5% silt, 1.4% clay). The contents of Cu, Ni, Cd, Pb, Zn, and Cr are summarized in Table 1. The contents of all PTEs (with the exception of Ni) exceeded the permissible values specified in the Ordinance of the Ministry of the Environment of Poland [25]. This was particularly visible in the case of Pb, where the permissible value was exceeded 20-fold, and Zn 8-fold.

### 3.2. Accumulation of PTEs in Soil After Experiment

**Figure 2:**
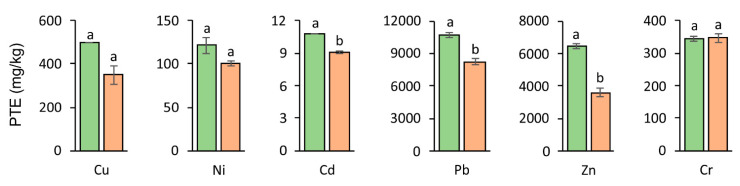
Accumulation of PTEs in soil after the experiment (■ Control ■ nZVI). For a given PTE, different letters indicate significant differences in PTE content in control soil and nZVI-amended soil.

The most dangerous factor in terms of ecology is the increased PTE concentration in the near-surface layer of the soil due to the danger of transfer via the root system of plants to their aboveground parts [31]. This, in turn, creates the risk of easy penetration to further links of the food chain. When applying nZVI to various remediation methods, attention should be drawn to the fact that the iron oxide shell enables the sorption and surface complexation of PTE ions, playing a key role in the immobilization of PTEs [32]. In the studies that were carried out, the addition of nZVI significantly influenced the concentration of individual PTEs in the soil following the harvest of test plants (Figure 2, Table 2). The application of nZVI is most effective in reducing the concentration of Zn in the soil; in the case of this element, its concentration decreased nearly two-fold compared to the control series. A similar relationship, though to a lesser extent, was noted in the case of Cu, Ni, and Pb, where the average content of individual elements under the influence of nZVI was also lower in relation to the control variant (without additives). nZVI reveals the ability to both reduce as well as adsorb elements [33]. The mechanism of reducing PTE ions remaining in contact with nZVI can take the course of two separate processes. The first relies on their reduction from the resulting direct contact with nZVI particles, whereas the second relies mainly on the adsorption of PTEs on the surface of the nZVI structure [34]. In the case of most PTEs, there is a possibility of their occurrence on intermediate oxidation levels which gradually undergo reduction to an oxidation state of zero [35].

### 3.3. Soil pH After Application on nZVI

**Figure 3:**
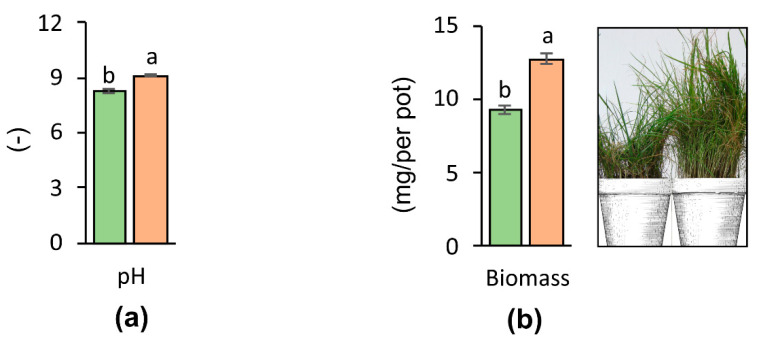
Soil pH (**a**) and plant biomass yield (**b**) (■ Control ■ nZVI). Different letters indicate significant differences in soil pH between control soil and nZVI-amended soil (a), and in biomass yield between control soil and nZVI-amended soil.

Many authors have drawn attention to the role of pH in taking up PTEs for plants and shaping their mobility in the soil [36,37,38]. When the pH is low, cation of PTEs forms, which influences the easy migration of these elements into the soil and facilitates their availability to plants [39]. However, in the case of alkaline soils, poorly soluble compounds with weak activity (which are only to a small extent available to plants), are formed [40]. In relation to the above, it seems well-based to apply soil additives which cause a significant increase in the pH value (F1,4 = 2414.3, *p* < 0.001). ZVI can easily be oxidized into amorphous iron oxy(hydr)oxides by O_2_ in soils and is accompanied by increases in soil pH (pH 8.0) due to the proton consumption [41]. The pH of soil used in the experiment is shown in Figure 3a. The nZVI applied in this study led to a significant increase in pH (by 0.88 unit) in relation to the control soil. This is confirmed by the studies of Bergano et al. [28] where the addition of nZVI to soil contaminated with As, Cu, Zn, and Pb increased soil pH values.

### 3.4. Effect of nZVI on the Plant Biomass Yield

PTEs contribute to changes in soil properties as well as negatively influence the development of plants and the biomass yield [42]. To overcome the negative effect of PTEs on plant growth, the use of suitable soil additives is important [43]. The influence of the addition of nZVI to soil strongly contaminated with PTEs on *Festuca rubra* L. and *Lolium perenne* L. biomass yield is presented in Figure 3b. The aboveground parts of the test plants in the controls (without the addition of nZVI) were characterized by a higher sensitivity to soil contamination with PTEs, as indicated by lower plant yield. The application of nZVI to pots resulted in a 38% rise in the yield of aboveground parts of plants compared to the control series (F1,4 = 116.36, *p* < 0.001). The plants subjected to the effects of nZVI show various effects, including the stimulation of sprouting or increased biomass [44,45]. As reported by Wang et al. [17], Trujillo-Reyes et al. [46], and Iannone et al. [47], oxidizing ZVI can create FeO particles, such as Fe_3_O_4_ and Fe_2_O_3_, which have a non-toxic or positive influence on the increase in growth of *Lolium perenne* L., *Lactuca sativa* L. and *Triticum aestivum* L. This result is in accordance with a previous report on nZVI-exposed Arabidopsis thaliana L., whose yield was found to be 38% higher in relation to the control series [48].

### 3.5. PTEs Accumulation in Aboveground Parts and Roots

**Figure 4:**
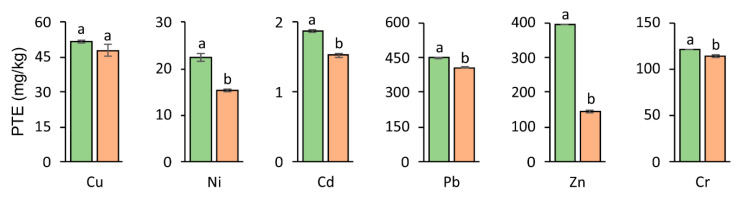
PTEs’ accumulation in aboveground biomass (■ Control ■ nZVI). For a given PTE, different letters indicate significant differences in PTE concentration in aboveground biomass between control soil and nZVI-amended soil.

**Figure 5:**
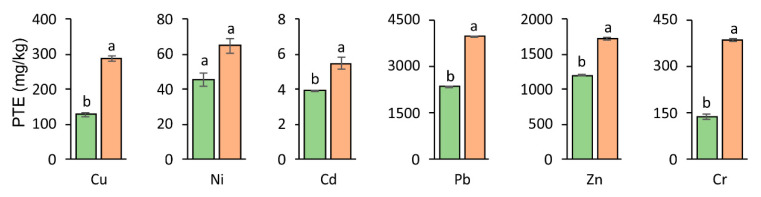
PTEs’ accumulation in roots (■ Control ■ nZVI). For a given PTE, different letters indicate significant differences in PTE concentration in roots between control soil and nZVI-amended soil.

In the phytostabilization technique, the process of immobilizing contaminants which occur in the soil takes place in the rhizosphere; this is in contrast to substances excreted in a natural manner by the roots which ensure that the microorganisms have an adequate amount of nutrients leading to the intensification of biological processes [49]. The total Cu, Ni, Cd, Pb, Zn, and Cr concentration was significantly higher in the roots than in the aboveground parts of the tested plant in pots with the addition of nZVI (Figure 4 and Figure 5, Table 2). It is known that root exudates can affect PTE availability in the rhizosphere [50,51]. They may also improve the reactivity of nZVI. As a result, the effectiveness of PTE stabilization can be improved and the PTE uptake by plants can be decreased. On the other hand, nanoparticles of ZVI can agglomerate in soil, leading to their uniform distribution and limited stabilization of PTEs [52]. The increase in PTE contents in plant roots can be explained by retaining iron particles in the apoplasts or in the outer layers of roots [53]. When analyzing the concentration of PTEs in the roots following the application of nZVI, it was found to be significantly higher (approximately two times higher) than its content in the aboveground parts in the case of Cu, Pb, and Cr. This relationship was confirmed in studies by Hidalgo et al. [54], who confirmed the highest Pb concentration in the roots of *Avicennia Germinans* L. The obtained results are also in accordance with the results of Gil-Diaz et al. [55], who showed that the addition of nZVI to soil contaminated with As significantly limited the accumulation of this element in the aboveground parts of *Hordeum vulgare* L.

## 4. Conclusions

The effect of nano zero valent iron (nZVI) on the efficiency of phytostabilization was investigated via pot experiments. The results showed that the addition of nZVI induced higher (38%) crop yields of the tested plants. The content of Cu, Ni, Cd, Pb, Zn, and Cr was significantly higher in the plant roots, with the effect intensified by the nZVI presence in the soil. This effect was particularly pronounced for Ni, Pb, and Zn following the application of nZVI, where the HM concentration was higher by 77%, 9-fold and 11-fold, respectively. Moreover, the pH of the soil amended with nZVI was significantly higher (by 0.88 per unit) than that of nonamended soil. In the case of soil, the addition of nZVI had the strongest influence on the reduction (46%) of its Zn contents in relation to the control series. Based on these results, we can conclude that the application of nZVI in the technique of aided phytostabilization to soils from sample post-industrial areas can have a significant influence on the protection and shaping of soil resources. Certain aspects, however, require further consideration in terms of nZVI application, e.g., other plant species, soil types, variable humidity conditions, and field test conditions. As a result, the use of nZVI can be widely used in processes supporting phytostabilization. However, the costs associated with nZVI production and its application, especially in large areas, may be higher compared to those of other mineral and organic amendments.

## Figures and Tables

**Table 1:** Total PTEs’ concentration in the soil used in the experiment.

Parameter	Unit	Value (Mean ± SD)	National Limit ^a^
Cu	mg/kg	**671.1** ± 78.5	600
Ni	mg/kg	129.3 ± 26.7	300
Cd	mg/kg	**22.4** ± 2.52	15
Pb	mg/kg	**13,479** ± 669.6	600
Zn	mg/kg	**8433** ± 1376.5	1000
Cr	mg/kg	**602.4** ± 11.4	500

^a^ Threshold concentration of PTEs in soils according to the regulations of the Polish Ministry of the Environment [25]. The values in bold are significantly above the threshold concentration.

**Table 2:** The results of Tukey’s HSD test for PTE contents in soil, aboveground biomass, and roots between control and nZVI.

Sample Type		Cu	Ni	Cd	Pb	Zn	Cr
Soil		0.07	0.18	0.003 **	0.02 *	0.01 *	0.87
Aboveground biomass		0.28	0.02 *	0.01 *	0.007 **	<0.001 ***	0.04 *
Roots		0.003 **	0.08	0.03 *	<0.001 ***	<0.001 ***	<0.001 ***
	* statistically significant difference at 0.05 significance level						
	** statistically significant difference at 0.01 significance level						
	*** statistically significant difference at 0.001 significance level						

## Data Availability

Data sharing is not applicable to this article.

## References

[1] Nascimento C.M., de Sousa Mendes W., Silvero N.E.Q., Poppiel R.R., Sayão W.M., Dotto A.C., Santos N.V., Amorim M.T.A., Demattê J.A.M. (2021). Soil degradation index developed by multitemporal remote sensing images, climate variables, terrain and soil attributes. J. Environ. Manag..

[2] Rodrigo-Comino J., López-Vicente M., Kumar V., Rodríguez-Seijo A., Valkó O., Rojas C., Pourghasemi H.R., Salvati L., Bakr N., Vaudour E. (2020). Soil science challenges in a new era: A transdisciplinary overview of relevant topics. Air Soil Water Res..

[3] Mazur Z., Radziemska M., Fronczyk J., Jeznach J. (2015). Heavy metal accumulation in bioindicators of pollution in urban areas of northeastern Poland. Fresenius Environ. Bull..

[4] Lebrun M., Miard F., Nandillon R., Hattab-Hambli N., Léger J.C., Scippa G.S., Morabito D., Bourgerie S. (2021). Influence of biochar particle size and concentration on Pb and As availability in contaminated mining soil and phytoremediation potential of poplar assessed in a Mesocosm Experiment. Water Air Soil Pollut..

[5] Liao S., Jin G., Khan M.A., Zhu Y., Duan L., Luo W., Jia J., Zhong B., Ma J., Ye Z. (2020). The quantitative source apportionment of heavy metals in peri-urban agricultural soils with UNMIX and input fluxes analysis. Environ. Technol. Innov..

[6] Tan K., Ma W., Chen L., Wang H., Du Q., Du P., Yan B., Liu R., Li H. (2021). Estimating the distribution trend of soil heavy metals in mining area from HyMap airborne hyperspectral imagery based on ensemble learning. J. Hazard. Mater..

[7] Radziemska M., Mazur Z., Fronczyk J., Jeznach J. (2014). Effect of zeolite and halloysite on accumulation of trace elements in maize (*Zea mays* L.) in nickel contaminated soil. Fresenius Environ. Bull..

[8] Gong Y., Zhao D., Wang Q. (2018). An overview of field-scale studies on remediation of soil contaminated with heavy metals and metalloids: Technical progress over the last decade. Water Resour..

[9] Tiodar E.D., Văcar C.L., Podar D. (2021). Phytoremediation and microorganisms-assisted phytoremediation of mercury-contaminated soils: Challenges and perspectives. Int. J. Environ. Res. Public Health.

[10] Xie L., van Zyl D. (2020). Distinguishing reclamation, revegetation and phytoremediation, and the importance of geochemical processes in the reclamation of sulfidic mine tailings: A review. Chemosphere.

[11] Hammond C.M., Root R.A., Maier R.M., Chorover J. (2020). Arsenic and iron speciation and mobilization during phytostabilization of pyritic mine tailings. Geochim. Cosmochim. Acta.

[12] Scattolin M., Peuble S., Pereira F., Paran F., Moutte J., Menad N., Faure O. (2020). Aided-Phytostabilization of steel slag dumps: The key-role of pH adjustment in decreasing chromium toxicity and improving manganese, phosphorus and zinc phytoavailability. J. Hazard. Mater..

[13] Wyszkowski M., Radziemska M. (2009). The effect of chromium content in soil on the concentration of some mineral elements in plants. Fresenius Environ. Bull..

[14] Zhan J., Huang H., Yu H., Zhang X., Zheng Z., Wang Y., Liu T., Li T. (2020). The combined effects of Cd and Pb enhanced metal binding by root cell walls of the phytostabilizer *Athyrium wardii* (Hook.). Environ. Pollut..

[15] Wetle R., Bensko-Tarsitano B., Johnson K., Sweat K.G., Cahill T. (2020). Uptake of uranium into desert plants in an abandoned uranium mine and its implications for phytostabilization strategies. J. Environ. Radioact..

[16] Li X., Yang Y., Gao B., Zhang M. (2015). Stimulation of peanut seedling development and growth by zero-valent iron nanoparticles at low concentrations. PLoS ONE.

[17] Wang H., Kou X., Pei Z., Xiao J.Q., Shan X., Xing B. (2011). Hysiological effects of magnetite (Fe_3_O_4_) nanoparticleson perennial ryegrass (*Lolium perenne* L.) and pumpkin (*Cucurbita mixta*) plants. Nanotoxicology.

[18] Gong X., Huang D., Liu Y., Zeng G., Wang R., Wan J., Zhang C., Cheng M., Qin X., Xue W. (2017). Stabilized nanoscale zerovalent Iron mediated cadmium accumulation and oxidative damage of *Boehmeria nivea* (L.) *Gaudich* cultivated in cadmium contaminated sediments. Environ. Sci. Technol..

[19] Jiang D., Zeng G., Huang D., Chen M., Zhang C., Huang C., Wan J. (2018). Remediation of contaminated soils by enhanced nanoscale zero valent iron. Environ. Res..

[20] Teodoro M., Clemente R., Ferrer-Bustins E., Martínez-Fernández D., Pilar Bernal M., Vítková M., Vítek P., Komárek M. (2020). Nanoscale zero-valent iron has minimum toxicological risk on the germination and early growth of two grass species with potential for phytostabilization. Nanomaterials.

[21] Gil-Díaz M., Pinilla P., Alonso J., Lobo M. (2017). Viability of a nanoremediation process in single or multi-metal (loid) contaminated soils. J. Hazard. Mater..

[22] Zand A.D., Tabrizi A.M., Heir A.V. (2020). The influence of association of plant growth-promoting rhizobacteria and zero-valent iron nanoparticles on removal of antimony from soil by *Trifolium repens*. Environ. Sci. Pollut. Res..

[23] Dong H., Deng J., Xie Y., Zhang C., Jiang Z., Cheng Y., Hou K., Zeng G. (2017). Stabilization of nanoscale zero-valent iron (nZVI) with modified biochar for Cr(VI) removal from aqueous solution. J. Hazard. Mater..

[24] Diego B., Rubén F., Lorena W. (2020). Nanoremediation of As and metals polluted soils by means of graphene oxide nanoparticles. Sci. Rep..

[25] Polish Ministry of the Environment (2016). Ordinance of the Minister of Environment on Soil and Ground Quality Standards.

[26] Radziemska M., Bęś A., Gusiatin Z.M., Cerda A., Mazur Z., Jeznach J., Kowal P., Brtnický M. (2019). The combined effect of phytostabilization and different amendments on remediation of soils from post-military areas. Sci. Total Environ..

[27] Baragaño D., Alonso J., Gallego J.R., Lobo M.C., Gil-Díaz M. (2020). Zero valent iron and goethite nanoparticles as new promising remediation techniques for As-polluted soils. Chemosphere.

[28] Baragaño D., Forján R., Fernández B., Ayala J., Afif E., Gallego J.L.R. (2020). Application of biochar, compost and ZVI nanoparticles for the remediation of As, Cu, Pb and Zn polluted soil. Environ. Sci. Pollut. Res..

[29] Pawluk K. (2015). Charakterystyka właściwości mechanicznych wybranych materiałów reaktywnych. Acta Sci. Pol. Archit..

[30] R Core Team (2020). R: A Language and Environment for Statistical Computing.

[31] Zhong X., Chen Z., Li Y., Ding K., Liu W., Liu Y., Yuan Y., Zhang M., Baker A.J.M., Yang W. (2020). Factors influencing heavy metal availability and risk assessment of soils at typical metal mines in Eastern China. J. Hazard. Mater..

[32] Mu Y., Jia F., Ai Z., Zhang L. (2016). Iron oxide shell mediated environmental remediation properties of nano zero-valent iron. Environ. Sci. Nano.

[33] Kim J.S., Shea P.J., Yang J.E., Kim J.E. (2007). Halide salts accelerate degradation of high explosives by zero-valent iron. Environ. Pollut..

[34] Lavine B.K., Auslander G., Ritter J. (2001). Polarographic studies of zero valent iron as a reductant for remediation of nitroaromatics in the environment. Microchem. J..

[35] Mukherjee R., Kumar R., Sinha A., Lama Y., Saha A.K. (2016). A review on synthesis, characterization and applications of nano zero valent iron (nZVI) for environmental remediation. Crit. Rev. Environ. Sci. Technol..

[36] Agrelli D., Caporale A.G., Adamo P. (2020). Assessment of the bioavailability and speciation of heavy metal(loid)s and hydrocarbons for risk-based soil remediation. Agronomy.

[37] Hijazin T., Radwan A., Lewerenz L., Abouzeid S., Selmar D. (2020). The uptake of alkaloids by plants from the soil is determined by rhizosphere pH. Rhizosphere.

[38] Romdhane L., Panozzo A., Radhouane L., Dal Cortivo C., Barion G., Vamerali T. (2021). Root characteristics and metal uptake of maize (*Zea mays* L.) under extreme soil contamination. Agronomy.

[39] Lu H.L., Nkoh N.J., Abdulaha-Al Baquy M., Dong G., Li J.Y., Xu R.K. (2020). Plants alter surface charge and functional groups of their roots to adapt to acidic soil conditions. Environ. Pollut..

[40] Gulio C., Camelin E., Tommasi T., Fino D., Pugliese M. (2020). Anaerobic digestates from sewage sludge used as fertilizer on a poor alkaline sandy soil and on a peat substrate: Effects on tomato plants growth and on soil properties. J. Environ. Manag..

[41] Qiao J., Liu T., Wang X., Li F., Lv Y., Cui J., Zeng X., Yuan Y., Liu C. (2018). Simultaneous alleviation of cadmium and arsenic accumulation in rice by applying zero-valent iron and biochar to contaminated paddy soils. Chemosphere.

[42] Bian F., Zhong Z., Zhang X., Yang C., Gai X. (2020). Bamboo—An untapped plant resource for the phytoremediation of heavy metal contaminated soils. Chemosphere.

[43] Cui H., Li H., Zhang S., Yi Q., Zhou J., Fang G., Zhou J. (2020). Bioavailability and mobility of copper and cadmium in polluted soil after phytostabilization using different plants aided by limestone. Chemosphere.

[44] Libralato G., Devoti C.A., Zanella M., Sabbioni E., Mičetić I., Manodori L., Pigozzo A., Manenti S., Groppi F., Ghirardini V.A. (2016). Phytotoxicity of ionic, micro- and nano-sized iron in three plant species. Ecotoxicol. Environ. Saf..

[45] Xie Y., Cheng W., Tsang P.E., Fang Z. (2016). Remediation and phytotoxicity of decabromodiphenyl ether contaminated soil by zero valent iron nanoparticles immobilized in mesoporous silica microspheres. J. Environ. Manag..

[46] Trujillo-Reyes J., Majumdar S., Botez C., Peralta-Videa J., Gardea-Torresdey J. (2014). Exposure studies of core-shell Fe/Fe_3_O_4_ and Cu/CuO NPs to lettuce (*Lactuca sativa*) plants: Are they a potential physiological and nutritional hazard?. J. Hazard. Mater..

[47] Iannone M.F., Groppa M.D., de Sousa M.E., van Raap M.B.F., Benavides M.P. (2016). Impact of magnetite ironoxide nanoparticles on wheat (*Triticum aestivum* L.) development: Evaluation of oxidative damage. Environ Exp. Bot..

[48] Yoon H., Kang Y.G., Chang Y.S., Kim J.H. (2019). Effects of zerovalent iron nanoparticles on photosynthesis and biochemical adaptation of soil-grown *Arabidopsis thaliana*. Nanomaterials.

[49] Zine H., Elgadi S., Hakkou R., Papazoglou E.G., Midhat L., Ouhammou A. (2021). Wild plants for the phytostabilization of phosphate mine waste in semi-arid environments: A field experiment. Minerals.

[50] Bravin M.N., Garnier C., Lenoble V., Gérard F., Dudal Y., Hinsinger P. (2012). Root induced changes in pH and dissolved organic matter binding capacity affect copper dynamic speciation in the rhizosphere. Geochim. Cosmochim. Acta.

[51] Kim K.R., Owens G., Kwon S. (2010). Influence of Indian mustard (*Brassica juncea*) on rhizosphere soil solution chemistry in long-term contaminated soils: A rhizobox study. J. Environ. Sci..

[52] Vítková M., Puschenreiter M., Komárek M. (2018). Effect of nano zero-valent iron application on As, Cd, Pb, and Zn availability in the rhizosphere of metal (loid) contaminated soils. Chemosphere.

[53] Martínez-Fernandez D., Komarek M. (2016). Comparative effects of nanoscale zerovalent iron (nZVI) and Fe_2_O_3_ nanoparticles on root hydraulic conductivity of *Solanum lycopersicum* L.. Environ. Exp. Bot..

[54] Hidalgo K.T.S., Carrion-Huertas P.J., Kinch R.T., Betancourt L.E., Cabrera C.R. (2020). Phytonanoremediation by *Avicennia germinans* (black mangrove) and nano zero valent iron for heavy metal uptake from Cienaga Las Cucharillas wetland soils. Environ. Nanotechnol. Monit. Manag..

[55] Gil-Diaz M., Diez-Pascuala S., González A., Alonso J., Rodríguez-Valdés E., Gallego J.R., Lobo M.C. (2016). A nanoremediation strategy for the recovery of an As-polluted soil. Chemosphere.

